# Ventilation efficacy during paediatric cardiopulmonary resuscitation (PEDIVENT): simulation-based comparative study

**DOI:** 10.3389/fmed.2024.1400948

**Published:** 2024-05-13

**Authors:** Tamara Skrisovska, Jana Djakow, Petr Jabandziev, Tereza Kramplova, Jozef Klucka, Martina Kosinova, Petr Stourac

**Affiliations:** ^1^Department of Paediatric Anaesthesiology and Intensive Care Medicine, University Hospital Brno and Faculty of Medicine, Masaryk University, Brno, Czechia; ^2^Department of Simulation Medicine, Faculty of Medicine, Masaryk University, Brno, Czechia; ^3^Paediatric Intensive Care Unit, NH Hospital Inc., Hořovice, Czechia; ^4^Department of Paediatrics, University Hospital Brno and Faculty of Medicine, Masaryk University, Brno, Czechia

**Keywords:** ventilation, paediatric, CPR, cardiopulmonary resuscitation, simulation

## Abstract

**Introduction:**

This simulation-based study aimed to evaluate the efficacy of ventilation during paediatric cardiopulmonary resuscitation (CPR) provided by healthcare professionals (HCPs) and lay rescuers (LRs). The objective was to assess the number of effective breaths delivered during the initial sequence of CPR. Effective ventilation plays a critical role during paediatric CPR as most cardiac arrests are secondary to hypoxia in origin. The recommendations on initial resuscitation in unresponsive, non-breathing children differ worldwide. The European Resuscitation Council (ERC) guidelines recommend five breaths before starting the chest compressions. Yet, this recommendation was based on the expert consensus historically and has not changed since 2000 because of the lack of evidence. This research addresses the identified knowledge gap, with potential implications for improving resuscitation practices and ultimately enhancing patient outcomes.

**Methods:**

HCPs and LRs performed 90 s of CPR involving two mannequins: 5-kg Baby and 20-kg Junior. Both groups (HCPs and LRs) performed the task before and after structured CPR training, and the efficacy of ventilation before and after the training was compared. The HCPs provided bag-mask ventilation; LR performed dispatcher-assisted CPR with mouth-to-mouth ventilation.

**Results:**

The number of participants that reached the primary outcome before and after the training in Baby was 26 (65%) vs. 40 (100%) in HCPs and 28 (60.9%) vs. 45 (97.8%) in LRs (improvement in both *p* < 0.001), respectively. The number of participants that reached the primary outcome before and after the training in the Junior mannequin was 31 (77.5%) vs. 32 (82.1%) in HCPs (*p* = 0.77) and 32 (82.1%) vs. 37 (94.9%) in LRs (*p* = 0.005), respectively.

**Discussion:**

This simulation-based study is the first to investigate ventilation efficacy during paediatric CPR provided by HCPs and LRs. Ventilation represents an important aspect of good-quality CPR in children. The concept of initiating paediatric CPR with initial breaths, as stated in ERC guidelines 2021, is justifiable. Trained HCPs and LRs providing dispatcher-assisted CPR could deliver effective ventilation to paediatric mannequins. These findings can contribute to future research in this area and address identified knowledge gaps concerning resuscitation guidelines, given the unique practical application of simulation as a research tool.

## 1 Introduction

Ventilation is an integral part of paediatric resuscitation, as recommended by the European Resuscitation Council (ERC), because oxygen depletion is the most common cause of cardiac arrest in children (1). Using chest compressions with rescue breaths is associated with higher survival to discharge and survival with a good neurological outcome to discharge than either no CPR or chest compression-only CPR in paediatric patients (2). Providing effective ventilation breaths in paediatric CPR is thus a parameter of good-quality CPR and has the potential to influence patient outcomes (3, 4). The weak, low certainty of evidence recommendation was raised for adult patients, suggesting that LRs who are trained, able, and willing to give rescue breaths and chest compressions should do so for all adult patients in cardiac arrest. Data were obtained from mannequins and training sessions (5). However, the data on the efficiency of ventilation during paediatric advanced life support (ALS) and basic life support (BLS) are lacking as it is difficult to establish the efficacy of ventilation in real-life resuscitation. This study aimed to close the knowledge gap with the unique application of simulation as a research tool.

The recommendation regarding the sequence of ventilation and compressions during paediatric CPR initial breaths during paediatric CPR varies worldwide (3, 6). The systematic review conducted by the ILCOR (International Liaison Committee on Resuscitation) 2020 (7) task force identified no difference in outcomes when comparing the sequence of paediatric CPR initiation regarding compressions–airway–breaths (CAB) as recommended by the American Heart Association (AHA) (3) guidelines versus airway–breaths–compressions (ABC) as stated in ERC (1) and Australian and New Zealand Committee on Resuscitation (ANZCOR) guidelines (6). In the latest version of ILCOR guidelines, the topic of the sequence of paediatric CPR was not addressed nor was the question of ventilation for LRs during paediatric CPR targeted (5).

ERC guidelines recommend starting ventilation with five initial breaths in paediatric CPR; this is based on expert opinion and is identified as a knowledge gap (1). The reasoning for initial breaths was the potential of reverting the hypoxia before the initiation of chest compressions as most of the paediatric cardiac arrests are secondary to hypoxia/asphyxia. Another reason for introducing five initial breaths was the possibility of reversing respiratory arrest with bradycardia (with a pulse) before the cardiac arrest occurs. The ANZCOR 2021 guidelines recommend paediatric CPR 30:2 (LRs) or 15:2 (HCPs) ratio starting with two breaths before initiating the chest compressions (6). The AHA guidelines did not differentiate between adults and children with regard to the initial approach in CPR and adopted a CAB sequence with a 30:2 ratio for lay rescuers and 15:2 for two or more HCPs (3). The reasoning for CAB is mainly uniformity and easier implementation as well as the shorter time needed for chest compression initiation. The ERC approach seems to be more pathophysiology-driven for children (and similarly, e.g., in the drowning or avalanche victims) (8) but having different adult and paediatric guidelines might make it harder to implement the system in the wider community. As there is no strong evidence favouring either sequence, it remains a controversial theme in the paediatric resuscitation community worldwide.

The analysis of breath effectiveness could provide information, clarify, and perhaps help unifying this discussed part of the resuscitation strategy. However, to the best of our knowledge, no study has ever analysed the effectiveness and quality of initial rescue breaths during simulated or real cardiopulmonary resuscitation of children and infants. The practical use of simulation as a research tool (9) may enable the closing of the identified knowledge gap during paediatric CPR. Due to ethical considerations, no other research modality applies to data collection. Other simulation-based studies have already been implemented as a source of data in the field of CPR (10, 11).

## 2 Materials and methods

### 2.1 Study design

In this prospective simulation-based study, we evaluated ventilation efficacy during simulated paediatric between HCPs, providing advanced life support (ALS) and LR’s delivering dispatcher-assisted basic life support (BLS) with mouth-to-mouth ventilation. The primary outcome was the number of ventilations (defined as a visible chest rise) out of the first five attempts in each group before structured training. We expected HCPs to achieve superior ventilation efficacy due to regular training than LRs, with at least four effective breaths out of five attempts for HCPs and at least three effective breaths out of five attempts for LRs. Secondary outcomes included analysing the first two ventilation attempts and assessing the distribution of low, ideal, and high volumes during CPR ventilation.

### 2.2 Settings

After receiving the letter of acknowledgement from the Ethics Committee of the University Hospital Brno, this study was performed at the Department of Simulation Medicine of the Medical Faculty of Masaryk University in the Simulation Centre of the Medical Faculty of Masaryk University (SIMU) and the Department of Paediatric Anaesthesiology and Intensive Care Medicine, Medical Faculty of Masaryk University, University Hospital Brno between April and July 2022. This study was registered on ClinicalTrials.gov (ID: NCT05345704, 26 April 2022).

The purpose of the study was explained to participants, and written informed consent was provided; participants’ characteristics were recorded: age, gender, role (physician/nurse/lay rescuer) and experience with real-life CPR (BLS/ALS/none). Two groups of possible rescuers were included: the healthcare professionals (HCPs) and the lay rescuers (LRs).

The presented case in both groups was a non-breathing paediatric patient, and the task was to perform a 90-s high-quality CPR, starting with five initial breaths and continuing with chest compression to ventilation ratio of 15:2 on two different simulation mannequins: a 5-kg infant (Baby) and a 20-kg child (Junior). The HCPs provided bag-mask ventilation (BMV), and the LRs provided mouth-to-mouth ventilation. BMV is utilised by HCPs in hospital settings, being an integral component of immediate resuscitation equipment. It is a primary device for ventilation during advanced life support (ALS). BMV is a skill trained among HCPs that provides care for potentially critically ill children. Therefore, we decided to set the scenario with BMV for the HCPs as a rational choice for enhancing the realism simulation scenario of in-hospital paediatric cardiac arrest with alignment to ALS initiation. No other equipment than the correct size mask and bag valve mask was available according to the scenario, which is often a situation of hospital arrest when CPR is initiated without pharmacotherapy at the department before the arrival of the medical emergency team.

**Figure 1:**
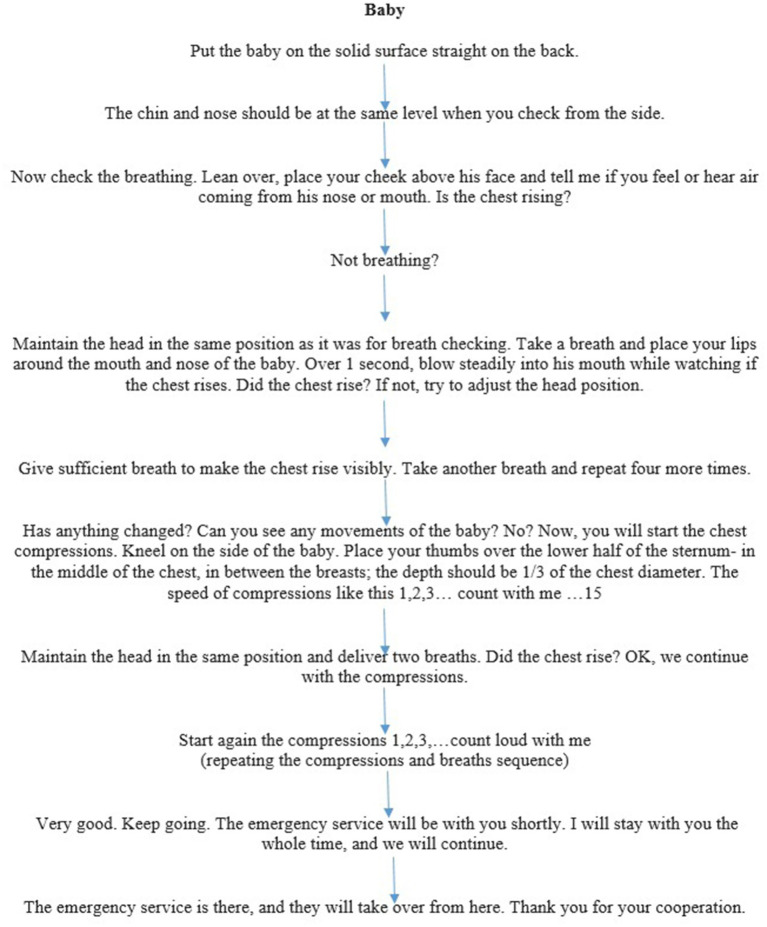

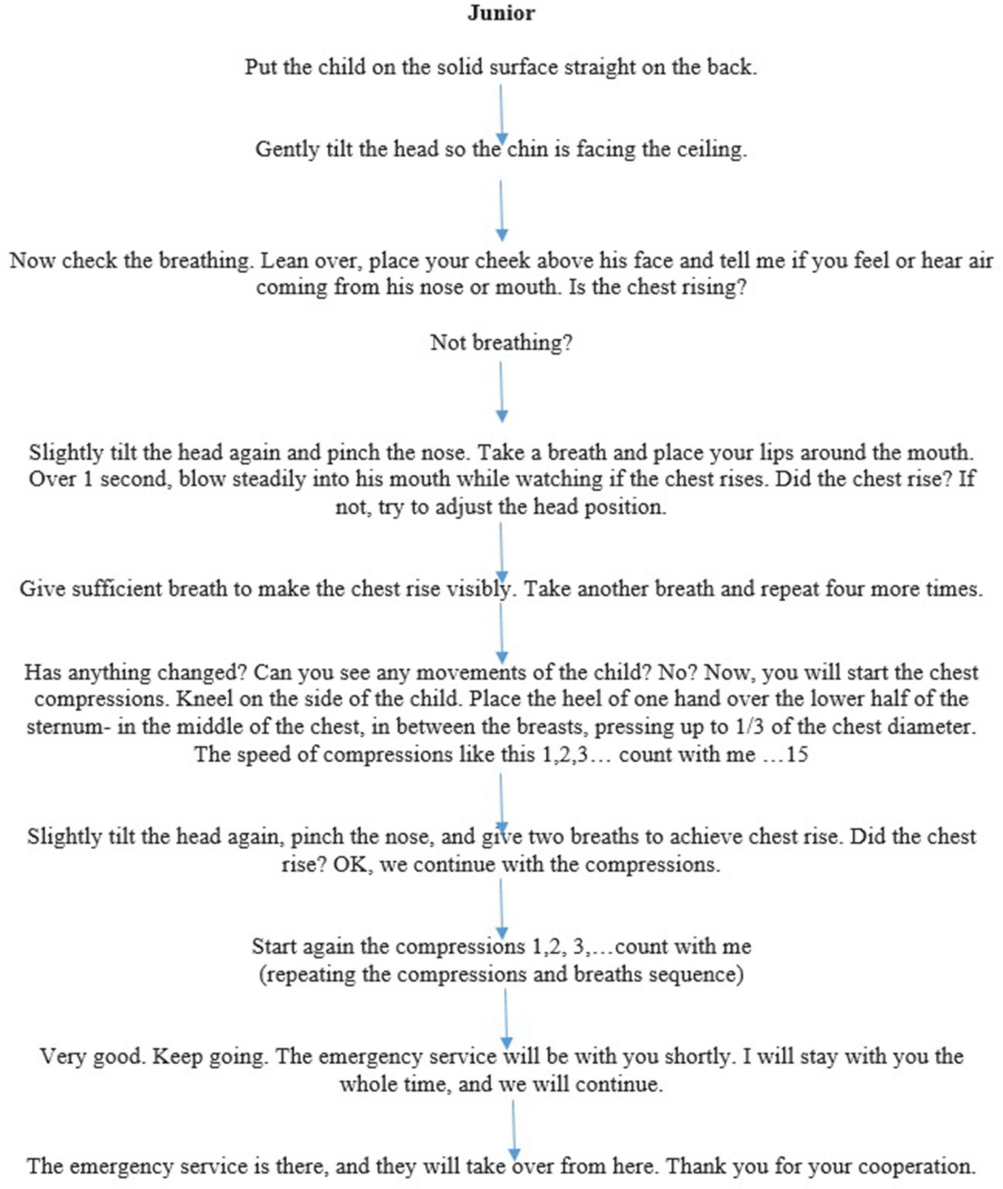
**(A)** Dispatcher-assisted basic life support (BLS) flowchart for a baby mannequin. **(B)** Dispatcher-assisted basic life support (BLS) flowchart for junior mannequin.

Conversely, in most basic life support (BLS) scenarios, LRs primarily rely on mouth-to-mouth ventilation, as they are typically not trained in using BMV, nor is such equipment readily accessible to them. The resuscitation in the group of LRs was led according to a standardised dispatcher-assisted CPR script provided to us by an authentic EMS dispatcher (Figure 1A Dispatcher-assisted basic life support flowchart for Baby and Figure 1B Dispatcher-assisted basic life support flowchart for Junior mannequin) after Emergency Medical Service (EMS) activation.

The ERC guideline algorithm 2021 of Paediatric Life Support was considered the standard for resuscitation in both groups (1). The ratio 15:2 for a dispatcher-assisted BLS was chosen to compare CRP-related measurements between HCPs and LRs better. The cases of paediatric cardiac arrest were presented and supervised by two certified European Paediatric Advanced Life Support (EPALS) instructors.

### 2.3 Participants

The HCP was trained and licenced to provide medical care and services to patients. In this study, we recruited physicians and nurses working at the paediatrics department (non-intensive care unit personnel) on a volunteer basis at Tertiary Children’s University Hospital, Brno. No further exclusion criteria were applied. HCPs are trained regularly once per year (mandatory in-hospital training) in CPR, which consists of a short theory introduction and individual training in high-quality CPR on Baby and Junior mannequin with direct feedback from the educator based on the data from the mannequin. Each training session was for 10 HCPs and lasted 75 min.

The second group were lay rescuers, recruited on a volunteer basis after the random call for participation in the CPR simulation-based study in high school visitors of the simulation centre. Anyone not considered a healthcare professional was acknowledged as a lay rescuer. No further exclusion criteria were applied.

After the first round of CPR on Baby and Junior mannequin that took place without educating the LRs in paediatric CPR, LRs underwent standardised training from two EPALS instructors, including a short theoretical introduction on causes of paediatric cardiac arrest, approach to the paediatric patient in cardiac arrest with the emphasis on effective ventilation, and high-quality chest compression with minimal flow time. After the theoretical part, there was one-to-one training in CPR on Baby and Junior mannequins, including correct head positioning, airway opening, effective ventilation recognition, correct rate, depth, and release of chest compressions. The training included 20 participants and lasted 75 min. After this training, we collected another set of data on Baby and Junior from the same group of LRs.

### 2.4 Data measurement

The measurements were obtained using two commercially available mannequins.

The infant mannequin was Laerdal Resusci Baby QCPR (Baby). The estimated weight of the simulated patient was declared to be 5 kg. The mannequin firmware version was 1.9.1.126.

The child mannequin was Laerdal Resusci Junior QCPR (Junior). The estimated weight of the simulated patient was declared to be 20 kg. The mannequin firmware version was 1.21.1.124.

Based on the latest information of the distributor at the time of study revision, there have been no significant firmware changes in the mentioned mannequins, ensuring study replicability.

Effective breath was defined as a visible chest rise, evaluated by the mannequin software and trained observers. To be deemed effective, the recording of the mannequin and assessment of the observer had to align. If the mannequin detected a breath without a visible chest rise (low-volume breath), it was not marked as effective. No discrepancies were recorded, as observers marked attempts as effective only when the mannequin recorded them. Secondary outcomes included the following: a sub-analysis of two first ventilation attempts, number and percentage of the five initial breath attempts with ideal, low, and high tidal volumes.

The ideal tidal volume was between 30 and 50 mL for the Baby (6 to 10 mL/kg). It was low if less than 30 mL volume was delivered. It was high if more than 50 mL volume was delivered.

The ideal tidal volume was between 120 and 200 mL for the Junior (6 to 10 mL/kg). It was low if less than 120 mL volume was delivered. It was high if more than 200 mL volume was delivered.

### 2.5 Bias

To avoid sampling bias, we used self-selection sampling as both HCPs and LRs participated on a volunteer basis. We piloted the measurement of visible chest rise between two independent researchers to ensure agreement in observation and data recording. Data about breath volumes were collected from the mannequin software.

### 2.6 Statistical methods

A power analysis determined sample sizes. The power analysis was based on the following prerequisites: A sample size of 40 achieves 80% power to detect a mean of paired differences of −0.8 with an estimated standard deviation of differences of 1.8 and a significance level (alpha) of 0.05 using a two-sided paired *t*-test.

Standard descriptive statistics were applied in the analysis: absolute and relative frequencies for categorical variables and mean and median continuous variables. The intra-individual differences before and after training were tested using the Wilcoxon signed-rank test. The level of statistical significance used in all tests was α = 0.05. Statistical analysis was computed using SPSS 28.0.1.1 (IBM Corp., 2021, IBM SPSS Statistics for Windows, Version 28.0.0. 1, Armonk, NY, United States).

## 3 Results

### 3.1 Participants

**Figure 2:**
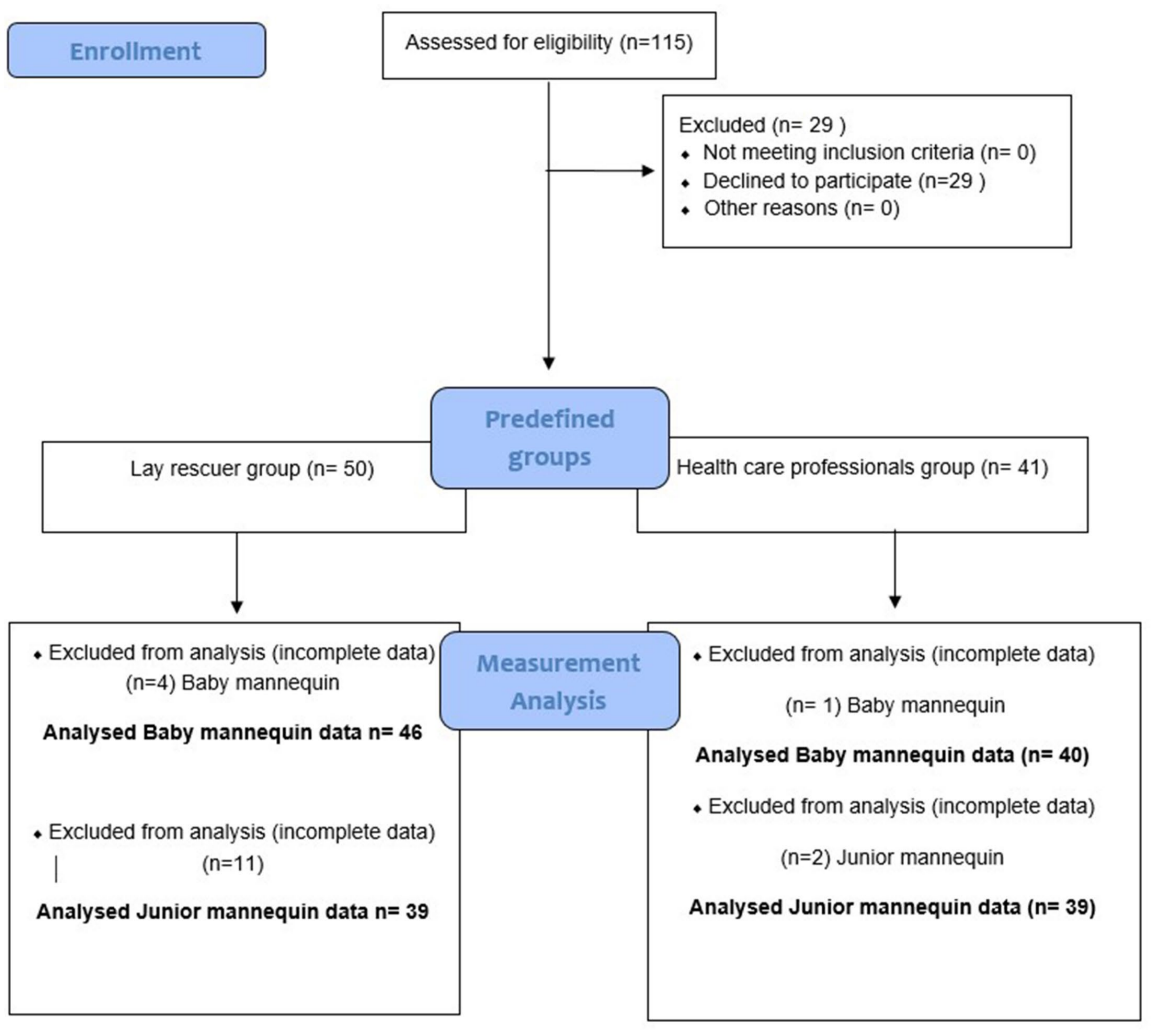
Flowchart of data acquisition.

There were, in total, 86 participants included in the analysis, of which 40 participants were healthcare professionals, and 46 were lay rescuers (see Figure 2 Flowchart of data acquisition). The reason for incomplete data was technical difficulties with the mannequins in HCPs and LRs.

### 3.2 Descriptive data

Participants’ demographics are displayed in Table 1.

**Table 1:** Participants’ demographics.

Characteristics	Lay rescuer group (*n* = 46)	Healthcare professional group (*n* = 40)
Woman	35 (76.1%)	35 (87.5%)
Age	16.8 (1.0)	41.3 (11.5)
Physicians	Not applicable	19 (47.5%)
Nurses	Not applicable	21 (52.5%)
CPR in real life (ALS)	Not applicable	23 (57.5%)
CPR in real life (BLS)	2 (4.3%)	0
Data not provided	0	1 (2.5%)

Data are presented as value (percentage), mean (standard deviation). CPR, cardiopulmonary resuscitation; ALS, advanced life support; BLS, basic life support.

### 3.3 Main results

#### 3.3.1 Primary outcome healthcare professionals

Four or more effective breaths out of five initial breath attempts (primary outcome for healthcare professionals) in the Baby mannequin were delivered by 26 HCPs (65%) before the training and 40 HCPs (100%) after the training, indicating a significant improvement in the number of effective ventilation attempts delivered before and after the training (*p* < 0.001). In the Junior mannequin, the primary outcome was reached by 31 HCPs (77.5%) before the training and 32 HCPs (82.1%) after the training; the improvement was not statistically significant (*p* = 0.77).

#### 3.3.2 Primary outcome lay rescuers

Three effective breaths out of five initial breath attempts (primary outcome for lay rescuers) in the Baby mannequin were delivered by 28 LRs (60.9%) before the training and 45 LRs (97.8%) after the training, the difference in the number of effective breaths before and after the training was statistically significant (P<0.001). In the Junior mannequin, the primary outcome was achieved by 32 LRs (82.1%) before the training and 37 LRs (94.9%) after the training, indicating an improvement in the number of effective breaths before and after the training (*p* = 0.005).

#### 3.3.3 Sub-analysis of the two first ventilation attempts Baby mannequin

In the Baby mannequin, the sub-analysis of the two first attempts revealed that in the HCP group, 24 participants (60.0%) were able to deliver effective breaths with their two ventilation attempts before the training and 39 HCPs (97.5%) after the training (*p* < 0.001).

In the lay rescuer group, 23 participants (50.0%) were able to deliver two effective breaths with their first two ventilation attempts before the training and 44 (95.7%) after the training; data from one LR participant were not valid (p < 0.001). Data are displayed in Table 2.

**Table 2:** Number of participants delivering 0 to 2 effective breaths out of the first 2 ventilation attempts.

Participant group	0 EB out of first two attempts *n* (%)	1 EB out of two first attempts *n* (%)	2 EB out of 2 first attempts *n* (%)
LR Baby BT	20 (43.5%)	3 (6.5%)	23 (50%)
LR Baby AT	0	1 (2.2%)	44 (95.7%)
HCP Baby BT	11 (27.5%)	5 (12.5%)	24 (60%)
HCP Baby AT	0	1 (2.5%)	39 (97.5%)
LR Junior BT	5 (12.8%)	7 (17.9%)	27 (69.2%)
LR Junior AT	0	0	38 (97.4%)
HCP Junior BT	5 (12.5%)	7 (17.5%)	28 (70%)
HCP Junior AT	3 (7.7%)	5 (12.8%)	31 (79.5%)

Participant group (lay rescuer = LR/healthcare professional = HCP), type of mannequin (Baby/Junior), before training (BT)/after training (AT), *n* = number of participants delivering 0–2 effective breaths out of first 2 ventilation attempts, percentage EB = effective breath. Note that data from one LR in Baby mannequin AT and one LR in Junior mannequin are missing due to technical issues.

#### 3.3.4 Sub-analysis of the two first ventilation attempts Junior mannequin

In the Junior mannequin, the sub-analysis of 2 initial ventilation attempts, 28 HCP participants (70.0%) were able to deliver two effective breaths with their first two ventilation attempts before the training, and 31 participants (79.5%) after the training (*p* = 0.308).

In the LRs group, 27 participants (69.2%) were able to deliver two effective breaths with their first two ventilation attempts before the training and 38 (97.4%) after the training (*p* = 0.02).

Overall effectivity during five initial ventilation attempts in Baby and Junior, before training (BT) and after training (AT) in both groups, is summarised in Table 3.

**Table 3:** Number of participants delivering 0 to 5 effective breaths out of the first 5 ventilation attempts.

Participant group	*n*	0	1	2	3	4	5
LR Baby BT	46	16 (34.8%)	1 (2.2%)	1 (2.2%)	3 (6.5%)	5 (10.9%)	20 (43.5%)
LR Baby AT	46	1 (2.2%)	0	0	0	0	45 (97.8%)
LR Junior BT	39	2 (5.1%)	2 (5.1%)	3 (7.7%)	3 (7.7%)	6 (15.4%)	23 (59.0%)
LR Junior AT	39	0	0	1 (2.6%)	0	3 (7.7%)	34 (87.2%)
HCP Baby BT	40	4 (10.0%)	5 (12.5%)	1 (2.5%)	4 (10.0%)	4 (10.0%)	22 (55.0%)
HCP Baby AT	40	0	0	0	0	0	40 (100%)
HCP Junior BT	40	2 (5.0%)	1 (2.5%)	1 (2.5%)	5 (12.5%)	4 (10.0%)	27 (67.5%)
HCP Junior AT	39	1 (2.6%)	2 (5.1%)	2 (5.1%)	2 (5.1%)	3 (7.7%)	29 (74.4%)

Participant group (lay rescuer = LR/healthcare professional = HCP), type of mannequin (Baby/Junior), before training (BT)/after training (AT), *n* = number of participants delivering 0–5 effective breaths out of first 5 ventilation attempts, percentage.

#### 3.3.5 Ideal, low, and high breath volumes

**Figure 3:**
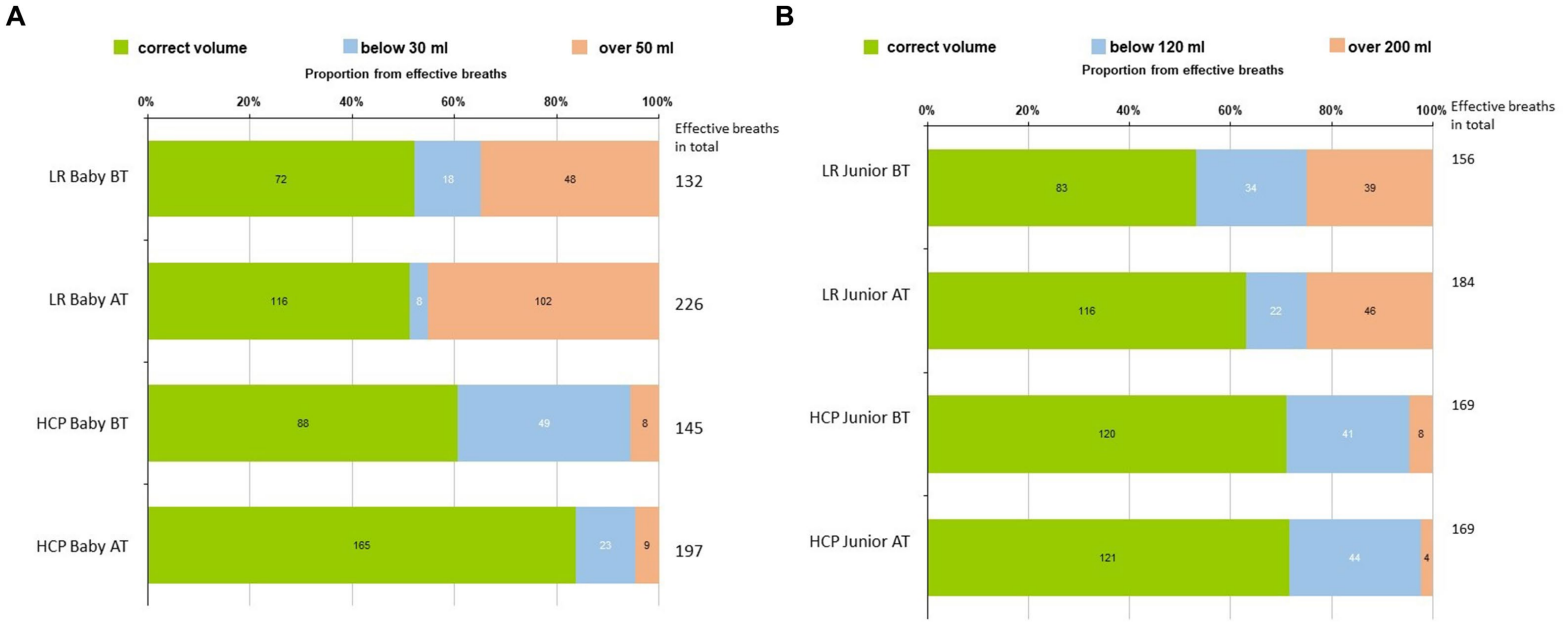
**(A)** Number and percentage of breaths with ideal (30-50 mL), low (below 30 mL), and high (over 50 mL) volumes from 5 initial breaths in the Baby mannequin. **(B)** The number and percentage of breaths with ideal (120–200 mL), low (below 120 mL), and high (over 200 mL) volumes from five initial breaths in the Baby mannequin.

The number and percentage of breaths with ideal (6–10 mL/kg), low (below 6 mL/kg), and high (over 10 mL/kg) tidal volume from five initial breaths are displayed in two tables: for Baby (Figure 3A). The number and percentage of breaths with ideal (30–50 mL), low (below 30 mL), and high (over 50 mL) tidal volume from effective ventilation attempts in Baby mannequin and for Junior (Figure 3B The number and percentage of breaths with ideal (120–200 mL), low (below 120 mL), and high (over 200 mL) tidal volume from five initial breaths in the Junior mannequin).

## 4 Discussion

### 4.1 Key results

The study suggests that trained healthcare professionals and lay rescuers providing dispatcher-assisted CPR could effectively administer initial breaths for infants and children. This implies that lay rescuers will likely provide effective breaths in real-life situations too. These findings support the ERC guidelines advocating for the initiation of paediatric CPR with initial breaths, given the potential reversal of the hypoxic cause underlying most paediatric cardiac arrests.

### 4.2 Interpretation

The data about ventilation volumes during CPR are limited. With the presented figures (Figures 3A,B), we would like to highlight the value of simulation-based training, particularly evident in the lay rescuers (LRs) group. In particular, the number of effective breaths before and after training increased in the Baby mannequin, rising from 132 before training to 226 after training within the same timeframe. Among HCPs, we observed an increase in correctly delivered volume breaths in the Baby mannequin before and after training. This improvement can be attributed to the emphasis on teaching the appropriate ventilation technique, which includes terminating ventilation upon visible chest rise to prevent overinflating. Proficiency in adequate ventilation is a skill that requires training. In the LR group, we noted an increase in high-volume breaths post-training, indicating a tendency towards overinflating during ventilation attempts. This phenomenon may be attributed to heightened stress levels among LRs, making it more challenging to provide correct ventilation and terminate it promptly upon chest rise, particularly with mouth-to-mouth techniques compared to bag-mask ventilation (BMV). Both hypoventilation and hyperventilation during CPR are associated with poorer outcomes (12), and thus, effective ventilation with adequate volumes should be addressed during regular CPR training in the same manner as the quality of chest compressions.

In Junior, there was an improvement in LRs regarding correct breath volumes by increasing the number of effective breaths (156 before training vs. 184 after training) and decreasing the number of low-volume breaths. The HCPs performed similarly before and after the training. The overall perception is that providing ventilation for older kids is technically easier than the same task on the Baby.

Participants without regular paediatric CPR training showed a steep learning curve, emphasising the importance of consistent practice. In particular, lay rescuers showed greater improvement than healthcare professionals with annual CPR training, underscoring the potential of simulation-based training for skill retention, as visible in the Junior mannequin.

Our study adds to future research in this field, addressing knowledge gaps in resuscitation guidelines using simulation as a practical research tool.

Simulation is now a widely used and well-established educational strategy in healthcare. It has also gained an important role as an investigation method in resuscitation-based research, allowing CPR quality checks and improvement, acknowledged in ERC guidelines (13).

The aim was to create a safe learning environment by keeping all actions confidential, encouraging participants to ask questions, and not being afraid to try CPR practically. Structured training was led while considering the specifics of adult learning theory using real-world learning examples, hands-on experience, and deliberate practice (14). The improvement in ventilation was more pronounced in the Baby mannequin. This may be due to the initial general concerns of the rescuers not to harm the baby when performing CPR. After the training, the participants become more confident in their skills and the potential benefits.

The topic of knowledge and skill retention after simulation-based training was discussed in studies, focusing mainly on compressions and using an automated external defibrillator (AED) (15). Past research has demonstrated significant CPR skill deterioration within 3 to 6 months, with maximal decline occurring within the first year after BLS training (16). This highlights the necessity for regular, instructor-led CPR training with structured feedback (17). Tailored training accommodating various learner types and styles is crucial for ensuring resuscitation knowledge and skill acquisition among HCPs and LRs. Participants with CPR training experience are more likely to perform bystander CPR, potentially impacting CPR outcomes (18). Data regarding ventilation skills retention in paediatric CPR training were not yet addressed and represent an opportunity for future research.

### 4.3 Limitations

Due to technical difficulties with the Junior mannequin, we could not collect data from all lay rescuers who performed CPR on this mannequin. Nevertheless, the sample size remained sufficient to fulfil the criteria of the power analysis, and, in our opinion, this only partially affected the overall results. The length of simulation scenario may also be a limitation; the main focus of our study was on five initial breaths. We have collected data about ventilation also after compression initiation, which we plan to use in the future investigations of ventilation during paediatric CPR, though the analysis was beyond the scope of this study. Another limitation lies in the primary outcome settings, as we aimed to differentiate between HCPs trained in ventilation during CPR and LRs lacking CPR experience, considering that not all breaths may prove effective. Consequently, we established a benchmark of at least four out of five attempts being effective for HCPs (80%) and three out of five attempts for LRs (60%) as our primary outcome. The objective was to assess initial breath delivery efficacy in both groups and their success following structured simulation-based training. This evaluation could enrich the discussions advocating for guidelines that incorporate initial breaths for paediatric CPR to potentially reverse hypoxemia as the most common cause for cardia arrest in children.

### 4.4 Generalisability

Because the study was conducted on simulation mannequins, extrapolating the findings to real-life situations is not straightforward and adds to the limitations of this study. Simulation is not intended to replace real clinical experience. While performing CPR in a simulation centre or *in situ* can mimic real CPR to a certain degree, it is important to note that stress levels during actual CPR performance may be higher compared to the simulation setting. In real-life situations, there can be other factors that may affect the willingness to deliver breaths like vomit or blood in the airway, although this may be more pronounced in adult patients and bystanders, rather than parents or family members. Conversely, conducting a similar study in real life would be highly challenging due to the overall low incidence of paediatric cardiac arrests and ethical concerns.

## 5 Conclusion

This study represents the first investigation of ventilation efficacy during simulated paediatric CPR, involving 40 healthcare professionals (HCPs) and 46 lay rescuers (LRs). The results show that both HCPs and LRs can deliver effective initial ventilation breaths during simulated paediatric CPR, with the implication that this could also be true for the real-life scenario; it also supports the efficacy of dispatcher-directed CPR. Consequently, the adoption of initiating paediatric CPR with initial breaths as in ERC guidelines, considering its hypoxic origin in most cases, appears to be a justifiable approach.

## Data availability statement

The raw data supporting the conclusions of this article will be made available by the authors, without undue reservation.

## Ethics statement

The studies involving human participants were reviewed and approved by the Ethics Committee of the University Hospital Brno, Jihlavska 20, 62500, Brno, Czech Republic (Project number 44/22). The patients/participants provided their written informed consent to participate in this study.

## Author contributions

TS: Conceptualization, Formal analysis, Investigation, Writing – original draft, Writing – review & editing. JD: Conceptualization, Methodology, Supervision, Validation, Writing – original draft, Writing – review & editing. PJ: Data curation, Methodology, Supervision, Writing – original draft, Writing – review & editing. TK: Conceptualization, Data curation, Methodology, Writing – original draft, Writing – review & editing. JK: Writing – original draft, Writing – review & editing, Conceptualization, Formal analysis. MK: Conceptualization, Data curation, Methodology, Writing – original draft, Writing – review & editing. PS: Data curation, Funding acquisition, Methodology, Supervision, Validation, Writing – original draft, Writing – review & editing. JK: Conceptualization, Writing – original draft, Writing – review & editing, Formal analysis.

## References

[1] Van de Voorde P Turner NM Djakow J de Lucas N Martinez-Mejias A Biarent D. European resuscitation council guidelines 2021: Paediatric life support. Resuscitation. (2021) 161:327–87. doi: 10.1016/j.resuscitation.2021.02.015, PMID: 33773830

[2] Goto Y Maeda T Goto Y. Impact of dispatcher-assisted bystander cardiopulmonary resuscitation on neurological outcomes in children with out-of-hospital cardiac arrests: a prospective, Nationwide, population-based cohort study. J Am Heart Assoc. (2014) 3:e000499. doi: 10.1161/JAHA.113.000499 24785780 PMC4309039

[3] Topjian AA Raymond TT Atkins D Chan M Duff JP Joyner BL. Part 4: pediatric basic and advanced life support: 2020 American Heart Association guidelines for cardiopulmonary resuscitation and emergency cardiovascular care. Circulation. (2020) 142:S469–523. doi: 10.1161/CIR.0000000000000901 33081526

[4] Scholz SS Borgstedt R Menzel LC Rehberg S Jansen G. Evolution and current state of global research on paediatric resuscitation: a systematic scientometric analysis. Scand. J. Trauma Resusc. Emerg. Med. Prosinec. (2020) 28:90. doi: 10.1186/s13049-020-00780-3 PMC7488007 32912262

[5] Berg KM Bray JE Ng KC Liley HG Greif R Carlson JN. 2023 international consensus on cardiopulmonary resuscitation and emergency cardiovascular care science with treatment recommendations: summary from the basic life support; advanced life support; pediatric life support; neonatal life support; education, implementation, and teams; and first aid task forces. Theatr Res Int. (2024) 195:109992. doi: 10.1016/j.resuscitation.2023.109992 37937881

[6] The ARC Guidelines (2022). Australian Resuscitation Council. Available at: https://resus.org.au/the-arc-guidelines/

[7] Nolan JP Maconochie I Soar J Olasveengen TM Greif R Wyckoff MH. Executive summary 2020 international consensus on cardiopulmonary resuscitation and emergency cardiovascular care science with treatment recommendations. Circulation. (2020) 156:A1–A22. doi: 10.1016/j.resuscitation.2020.09.009, PMID: 33098915 PMC7576314

[8] Lott C Truhlář A Alfonzo A Barelli A González-Salvado V Hinkelbein J. European resuscitation council guidelines 2021: cardiac arrest in special circumstances. Resuscitation. (2021) 161:152–219. doi: 10.1016/j.resuscitation.2021.02.011, PMID: 33773826

[9] Cheng A Auerbach M Hunt EA Chang TP Pusic M Nadkarni V. Designing and conducting simulation-based research. Pediatrics. (2014) 133:1091–101. doi: 10.1542/peds.2013-3267 24819576

[10] Kosinová M Štourač P Prokopová T Vafková T Vafek V Barvík D. Feasibility of mouth-to-mouth ventilation through FFP2 respirator in BLS training during COVID-19 pandemic (MOVERESP study): simulation-based study. Children. (2022) 9:1751. doi: 10.3390/children9111751 36421199 PMC9688859

[11] Andriessen P Oetomo SB Chen W Feijs LM. Efficacy of feed forward and feedback signaling for inflations and chest compression pressure during cardiopulmonary resuscitation in a newborn mannequin. J Clin Med Res. (2012) 4:274–8. doi: 10.4021/jocmr865w 22870175 PMC3409623

[12] Aufderheide TP Sigurdsson G Pirrallo RG Yannopoulos D McKnite S von Briesen C. Hyperventilation-induced hypotension during cardiopulmonary resuscitation. Circulation. (2004) 109:1960–5. doi: 10.1161/01.CIR.0000126594.79136.61 15066941

[13] Greif R Lockey A Breckwoldt J Carmona F Conaghan P Kuzovlev A. European resuscitation council guidelines 2021: education for resuscitation. Resuscitation. (2021) 161:388–407. doi: 10.1016/j.resuscitation.2021.02.016 33773831

[14] Inott T Kennedy BB. Assessing learning styles: practical tips for patient education. Nurs Clin North Am září. (2011) 46:313–20. doi: 10.1016/j.cnur.2011.05.006, PMID: 21791266

[15] Woollard M Whitfield R Smith A Colquhoun M Newcombe RG Vetter N. Skill acquisition and retention in automated external defibrillator (AED) use and CPR by lay responders: a prospective study. Resuscitation. (2004) 60:17–28. doi: 10.1016/j.resuscitation.2003.09.006, PMID: 15002485

[16] Wilson E Brooks B Tweed WA. CPR skills retention of lay basic rescuers. Ann Emerg Med srpen. (1983) 12:482–4. doi: 10.1016/S0196-0644(83)80643-X, PMID: 6881644

[17] González-Salvado V Rodríguez-Ruiz E Abelairas-Gómez C Ruano-Raviña A Peña-Gil C González-Juanatey JR. Training adult laypeople in basic life support. a systematic review. Rev Esp Cardiol Engl. (2020) 73:53–68. doi: 10.1016/j.rec.2018.11.013 30808611

[18] Tanigawa K Iwami T Nishiyama C Sinceogi H Kawamura T. Are trained individuals more likely to perform bystander CPR? An observational study. Resuscitation. (2011) 82:523–8. doi: 10.1016/j.resuscitation.2011.01.027 21354688

